# Violence Against Paramedics: Protocol for Evaluating 2 Years of Reports Through a Novel, Point-of-Event Reporting Process

**DOI:** 10.2196/37636

**Published:** 2023-03-16

**Authors:** Justin Mausz, Elizabeth A Donnelly

**Affiliations:** 1 Peel Regional Paramedic Services Brampton, ON Canada; 2 School of Social Work The University of Windsor Windsor, ON Canada

**Keywords:** paramedic, emergency medical services, emergency, violence, harm, abuse, workplace violence, work, safety, occupational health, reporting, epidemiology, epidemiological, point-of-event: development, implementation, mixed methods

## Abstract

**Background:**

Violence against paramedics has been described as a serious public health problem with the potential for significant physical and psychological harm, but the organizational culture within the profession encourages paramedics to consider violence as just “part of the job.” Therefore, most incidents of violence are never formally documented. This limits the ability of researchers and policy makers to develop strategies that mitigate the risk and enhance paramedic safety.

**Objective:**

Following the development and implementation of a novel, point-of-event violence reporting process in February 2021, our objectives are to (1) estimate the prevalence of violence and generate a descriptive profile for incidents of reported violence; (2) identify potentially high-risk service calls based on characteristics of calls that are generally known to the responding paramedics at the point of dispatch; and (3) explore underpinning themes, including intolerance based on gender, race, and sexual orientation, that contribute to incidents of violence.

**Methods:**

Our work is situated in a single paramedic service in Ontario, Canada. Using a convergent parallel mixed methods approach, we will retrospectively review 2 years of quantitative and qualitative data gathered from the External Violence Incident Report (EVIR) system from February 1 2021 through February 28, 2023. The EVIR is a point-of-event reporting mechanism embedded in the electronic patient care record (ePCR) developed through an extensive stakeholder engagement process. When completing an ePCR, paramedics are prompted to file an EVIR if they experienced violence on the call. Our methods include using descriptive statistics to estimate the prevalence of violence and describe the characteristics of reported incidents (Objective 1), logistic regression modeling to identify high-risk service calls (Objective 2), and qualitative content analysis of incident report narratives to identify underpinning themes that contribute to violence (Objective 3).

**Results:**

As of January 1, 2023, 377 paramedics—approximately 1 in 5 active-duty paramedics in the service—have filed a total of 975 violence reports. Early analysis suggests 40% of reports involved a physical assault on the reporting paramedic. Our team is continuing to collect data with more fulsome analyses beginning in March 2023. Our findings will provide much-needed epidemiological data on the prevalence of violence against paramedics in a single paramedic service, its contributing themes, and potential risk factors.

**Conclusions:**

Our findings will contribute to a growing body of literature demonstrating that violence against paramedics is a complex problem that requires a nuanced understanding of its scope, risk factors, and contributing circumstances. Collectively, our research will inform larger, multisite prospective studies already in the planning stage and inform organizational strategies to mitigate the risk of harm from violence.

**International Registered Report Identifier (IRRID):**

DERR1-10.2196/37636

## Introduction

### Background

At the start of the COVID-19 pandemic, paramedics had some of the highest rates of posttraumatic stress injury (PTSI) among public safety personnel (PSP) in Canada. A recent cross-sectional survey of PSP in Canada found that 1 in 4 participating paramedics met the screening criteria for posttraumatic stress disorder (PTSD), 1 in 3 for major depressive disorder, and 1 in 3 for an anxiety disorder [1], with nearly half of the paramedics meeting the screening criteria for a current mental disorder [2]. Related research has also demonstrated high rates of exposure to trauma [3-5], disturbed sleep [6], adverse childhood experiences [7], chronic pain [2,8], and alarming rates of suicidality [9]—a situation that has since been described in research and policy as a “crisis in Canada” [10,11].

One dimension of the crisis that has received comparatively little attention is the exposure of paramedics to acts of intentional workplace violence. Although we know from previous research that situations involving threats to physical safety among PSP are associated with an increased risk of PTSI [3,5,12,13], the specific contribution of violence remains understudied. Part of the problem is that—in the absence of significant physical injuries—incidents of violence often go unreported. For example, in a 2014 survey of paramedics, over 75% disclosed having been subjected to workplace violence in the past calendar year. Despite these acts of violence negatively impacting their job satisfaction and home life, less than 20% of survey participants took any action to report the incidents to supervisors or the police [14]. This parallels a growing body of research internationally that indicates that violence against paramedics is a “serious public health problem” [15] that remains “vastly underreported” [16].

The organizational culture within the paramedic profession appears to contribute significantly to underreporting. In a qualitative study of paramedics in Ontario, Canada, we identified a complex interplay of organizational culture features in sustaining cultural norms that lead paramedics to downplay the significance of incidents and effectively position the ability for paramedics to “brush off” violent encounters as an expected professional competency [17]. Underreporting hinders the ability of researchers and policy makers alike to develop solutions to mitigate risk and enhance paramedic safety, as the scope of the problem, its contributing risk factors, and the potential harms remain poorly understood. Given the high rates of mental disorders among paramedics [1] and a growing hostility toward health care providers during the COVID-19 pandemic [18], the need for action has never been more apparent. Critical to intervening, however, is the need for robust epidemiological data to understand the problem more fully.

### The EVIR Process

The External Violence Incident Report (EVIR) (Multimedia Appendix 1) is a novel, point-of-event reporting process embedded in the electronic patient care record (ePCR) in Peel Regional Paramedic Services (PRPS) in Ontario, Canada. Developed in 2020 through an extensive gap analysis, stakeholder consultation, and pilot testing process [19], the EVIR was launched in February 2021. When completing an ePCR after a 911 service call, paramedics in Peel Region are prompted to complete an EVIR if they experienced any form of verbal abuse, intimidating behavior, sexual harassment, or physical or sexual assault during the call. Using a series of drop-down menus and free-text narrative boxes, the EVIR gathers information about the type of violence, its source, and contributing circumstances in addition to administrative data (eg, time of day, call type, patient acuity, etc) that automatically populate from the corresponding ePCR. The goal of developing the EVIR was to produce a reporting process that minimized the administrative burden on the reporting paramedics by leveraging technological efficiencies (eg, by auto populating relevant fields) and minimize the amount of manual free-text entries or nonrelevant fields. The result is an accessible, user-friendly, and culturally accepted means of reporting violent incidents in real time.

### Aim and Research Objectives

Following a year since the implementation of the EVIR, we aim to explore the prevalence of, risk factors for, and circumstances that contribute to incidents of reported violence against paramedics in Peel Region, Ontario, Canada. Using a convergent parallel mixed methods approach [20], our objectives are to (1) describe the prevalence and characteristics of violence against paramedics in the Peel Region; (2) explore features of 911 service calls associated with an increased risk of violence; and (3) qualitatively explore underpinning circumstances that contribute to incidents of violence, including harassment based on gender, race and related grounds, and sexual orientation.

## Methods

### Overview

**Figure 1:**
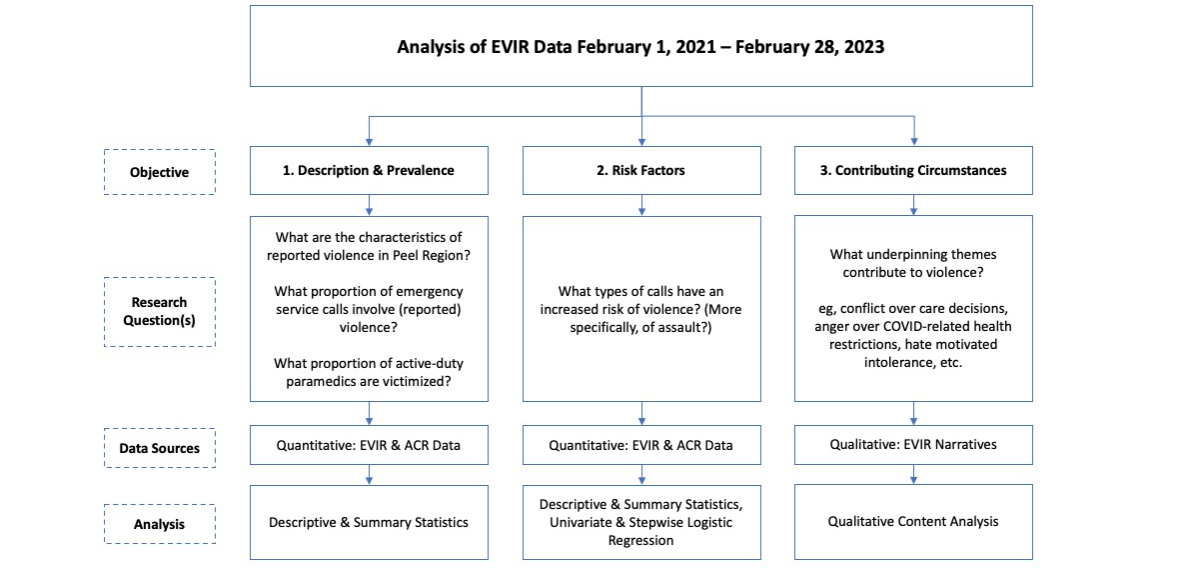
Overview of project objectives, research questions, data sources, and analysis plans. ACR: Ambulance Call Report; EVIR: External Violence Incident Report.

This program of research is situated within a realist ontology [21] and uses a convergent parallel mixed methods approach [20] to broadly explore the prevalence, risk factors, and circumstances that contribute to incidents of violence against paramedics. We provide an overview of our methods blueprinted for each research objective in Figure 1. In brief, our approach includes calculating descriptive and summary statistics to characterize the prevalence and features of reported violence (Objectives 1 and 3) and logistic regression modeling to explore risk factors (Objective 2). To address Objective 3, we will use qualitative content analysis [22] within an interpretivist epistemology [23] to identify and define themes that contribute to incidents of reported violence. Data for the research will be abstracted from completed EVIRs and other administrative data routinely gathered from ePCRs.

### Ethical Considerations

Research ethics review for this program was submitted to the University of Toronto Research Ethics Board (REB protocol number 44162). All data used in the research will be scrubbed of direct identifiers (eg, patient or paramedic names, addresses of call locations, and call identification numbers). Anonymized alphanumeric participant identification codes will be substituted for paramedic names and the original linkages destroyed after deidentification. We will work to ensure that any potential combinations of indirect identifiers (particularly narrative descriptions of incidents) are presented in such a way as to reduce the risk of reidentification to the greatest degree reasonably possible.

There are 4 participant groups to whom consent requirements apply (Table 1). The EVIR has an embedded passive consent process wherein reporting paramedics (Group C) who do not want the information contained in the report used for research purposes can opt out by checking a box. During training on the EVIR, paramedics in the service were made aware that although the EVIR is not specifically intended as a research intervention per se, data from the EVIR may be subsequently used for research purposes in much the same way that health records are used retrospectively in medical research. Because of the sensitive nature of the data, we informed paramedics of the ability to indicate a preference (via the opt-out mechanism) for the future use of a specific report. This affords the paramedics the ability—at the time of reporting—to dissent to the future use of the report for subsequent research purposes.

**Table 1:** Participant groups included in the analysis.

Group	Violent incident	No violent incident
Patient	A	B
Paramedic	C	D

The Tri-Council Policy on the Ethical Conduct for Research Involving Human Participants [24] sets out specific criteria under which alterations to the consent process can be contemplated: (1) where the research involves no more than minimal risk to the participants, (2) the alteration to the consent requirements is unlikely to adversely affect the welfare of the participants, (3) it is impossible or impractical to conduct the research if prior consent of the participants is required, and (4) the precise nature and extent of the alteration to consent is defined. We will seek to waive the requirement for informed participant consent from patients (Groups A and B) on the grounds of minimal risk, necessity, and impracticality. Particularly for Group A, seeking prior consent from alleged perpetrators of violence would pose legitimate safety risks to the investigators. The information gathered from Group B is minimal and contains no direct or indirect risk of identification. Finally, no data are being collected directly from Group D, and we do not anticipate any risk of direct or indirect identification or harm to nonreporting paramedics.

### Setting and Context

This research is situated in the Regional Municipality of Peel in Ontario, Canada. The PRPS is the publicly funded, sole provider of land ambulance paramedic services to this region, employing approximately 700 primary and advanced care paramedics (PCP/ACPs) [25]. Peel Region is divided geographically into 4 quadrants with 1 central paramedic headquarters facility per quadrant (called “divisions”). Paramedics report for work at 1 of the 4 divisions and are then deployed to several smaller community stations strategically located in a “hub and spoke” model. Crew configurations include PCP-PCP, ACP-PCP, and solo responder (of either classification) models on emergency response units. PRPS responds to over 130,000 emergency calls per year, making it the second-largest paramedic service organization in the province in terms of staffing and caseload. Documentation standards mandate the completion of an Ambulance Call Report (ACR) for any service call for which paramedics arrive on the scene. In 2019, the service launched the External Violence Against Paramedics working group with the 2-fold mandate of gathering data to develop evidence-informed strategies to mitigate the risk of violence and enhance paramedic safety.

In 2020, 1 year before the implementation of the EVIR, the service recorded a total of 60 documented incidents of injury resulting from workplace violence, with the type of violence and the requirement for injury reporting left to the discretion of the affected paramedic and their supervisor. Following the development of the EVIR, the service released a new policy for staff that outlines the roles and responsibilities of paramedics and service leadership in identifying, intervening in, and reporting incidents of violence. Importantly, the new policy specified the types of violence to be captured as part of the reporting process with definitions drawn from a review of the literature and relevant occupational health and safety legislation (Table 2).

**Table 2:** Definitions of incident types in the External Violence Incident Report (EVIR)^a^.

Type of violence	Definition
Verbal abuse	Offensive or hateful language; yelling or screaming with the intent of frightening the paramedic
Intimidation	Purposely threatening, following, or using gestures to offend or frighten the paramedic
Assault	Physical attack or attempt to attack; punching or kicking or using a weapon with the intent of causing bodily harm
Sexual harassment	Sexual propositioning or unwelcome sexual attention from a perpetrator. Humiliation, remarks, or offensive jokes with sexual overtones, suggestive looks, or physical gestures
Sexual assault	Indecent assault. Brushing, touching, or grabbing of genitals or breast area

^a^Adapted from Bigham et al [14] with language from the Ontario Occupational Health and Safety Act.

### Objective 1: Prevalence and Characteristics

#### Research Questions

Objective 1 is concerned with answering the following research questions: (1) What proportion of 911 service calls involve exposure to reported violence? (2) What proportion of active-duty paramedics report experiencing violence? (3) What are the characteristics (eg, type and source of violence, location, time of day, etc) of incidents of reported violence?

#### Data Sources

A detailed accounting of the variables included in this study is provided in Multimedia Appendix 2. We will abstract the following administrative data from all ACRs completed during the study period: paramedic station of origin (division), paramedic level of care (eg, PCP/ACP), date, call received time, pick-up location code, dispatch priority, dispatch problem code, primary problem code, and return priority.

Where an EVIR is completed for a particular service call, we will additionally abstract the following fields from the violence report: report creator, incident type, source of violence, location of the incident, contributing circumstances (eg, alcohol intoxication, drug use, cognitive impairment), police present (yes/no), police response helpful (yes/no; narrative), and address hazard flag (communicated, created/extended).

The data will be assembled in Microsoft Excel (Microsoft Corp). An anonymized alphanumeric participant code will be substituted for the report creator’s name by the data custodian.

#### Analyses

We will use descriptive and summary statistics to characterize the data, including means and medians with measures of dispersion for continuous data and counts and percentages for categorical data. Continuous variables will be plotted and assessed for normality, both visually and with skewness and kurtosis tests. We will then calculate the proportion of paramedics reporting exposure to violence and the average number of reports per paramedic. We will assess for group differences in reporting using 1-way ANOVA for continuous variables or chi-square for proportions, with significance set at *P*<.05. All analyses will be carried out in SPSS (version 28; IBM Corp).

### Objective 2: Risk Factors

#### Research Questions

Objective 2 is concerned with answering the following research questions: (1) What characteristics of emergency calls are associated with an increased risk of reported violence? (2) Relatedly, what characteristics of emergency calls are associated with an increased risk of a violent encounter resulting in any form of assault?

The goal here is to assess the degree to which characteristics of emergency calls that are typically known to the responding paramedics before arrival at the scene can be used to identify service calls that have a higher risk of violence. This information can be used to develop risk mitigation strategies, such as coordinated responses with paramedic supervisors, police, or mobile crisis intervention teams.

#### Data Sources

Data for Objective 2 will be drawn from the ACR database and completed EVIRs with all the same data points listed in Objective 1.

#### Analyses

Our outcomes of interest are (1) whether an EVIR is filed for a particular service call (“EVIR”; yes/no) and (2) whether a service call results in a reported assault on a paramedic. Because paramedics can select multiple types of violence on the EVIR (eg, verbal abuse, assault, and intimidation), the assault outcome will require collapsing the incident type categories into a new output variable of (“Assault_Paramedic”; yes/no) that captures incidents that include combinations of violence that involve either physical or sexual assault.

Potential covariates in our analysis will include service call characteristics that are typically known to the responding paramedics at the point of dispatch. These include the day of the week, time of day, call/pick-up location (26 levels, eg, residence, street, restaurant/bar), dispatch problem code (55 levels, eg, unconscious, drug/alcohol overdose, dyspnea), patient sex (male/female/other), and patient age.

Paramedics in Peel Region normally work 12-hour day or night shifts with additional staff scheduled during “peak” demand periods (broadly, 10 AM to 10 PM). However, in our analysis, we will define 3 nonoverlapping 8-hour “buckets” adapted from paramedic shift scheduling: day (6 AM to 1:59 PM), afternoon (2 PM to 9:59 PM), and overnight (10 PM to 5:59 AM).

Given the large number of variables, our modeling approach involves several preliminary steps to whittle down the list of covariates to be included. We will first calculate frequencies by running cross tabulations for each variable with our outcomes of interest. Levels of variables (eg, pick-up location, dispatch problem code) with fewer than 10 observed events will be removed. Next, we will run univariate logistic regression analyses for each variable level in descending order of observed proportion. Variables that are significantly associated with each outcome at a threshold of *P*<.10 will be retained for the next stage of model construction.

Our interest in constructing multivariate logistic regression models is in the adjusted odds ratios (AORs) of the individual covariates rather than the predictive capacity of the model as a whole. We will construct our adjusted models using a forward step approach in declining order of significance (ie, starting with the smallest *P* values) with the condition that all included variables must retain a *P*<.05 with AORs whose confidence intervals do not include the null value. Finally, we will test for interactions between the pick-up location code and the dispatch problem code, constructing terms based on the significance of individual covariates in univariate modeling and introducing terms sequentially to the adjusted models for evaluation. Interaction terms that do not meet the significance threshold will be removed from the model.

The total number of included covariates will also necessarily be constrained by the observed event rate; consequently, we will not exceed an event-to-covariate ratio of less than 10:1.

### Objective 3: Contributing Circumstances

#### Research Questions

Objective 3 is concerned with answering the following research questions: (1) What underpinning themes contribute to violence against paramedics? (2) Relatedly, what proportion of EVIRs involve harassment on grounds that are protected under the Ontario Human Rights Code (ie, gender, race/ethnicity, and sexual orientation)? (3) What is the potential contribution of conflict over COVID-19 health measures/restrictions to incidents of reported violence?

#### Data Sources

Each EVIR contains a space for the reporting paramedic to enter a narrative description in response to the “what happened?” prompt. During the rollout phase, paramedics were instructed to be detailed and specific in documenting the details of violent incidents and to include direct quotes where appropriate. We will abstract these free-text narratives for analysis.

#### Analyses

Approaching the data from an interpretivist epistemology [23,26], we will use qualitative content analysis [22,27] to answer each of our research questions. Qualitative content analysis is a technique for identifying themes in textual data using iterative, in vivo codes or the application of an a priori coding framework. As an analytical strategy, qualitative content analysis remains “close” to the surface of the data, avoiding the sort of deep inferences or abstractions that are germane to other qualitative methodologies (ie, grounded theory or ethnography). For Research Question 1, the incident narratives will be abstracted and imported into NVivo for Mac (QSR International) for analysis. We will inductively code the text using techniques described by Charmaz [28], with successive rounds of open [29] and then focused [30] coding to identify themes and then define categories based on conceptual similarity. This iterative approach will allow us to explore circumstances that underpin incidents of reported violence, such as conflict over patient care (eg, choice of hospital) that may escalate into violence.

For Research Question 2, we are guided by language in the Ontario Human Rights Code [31]. In brief, the law explains that all persons have the right to be free from discrimination and harassment based on 14 protected grounds. The code defines harassment as “engaging in a course of vexatious comment or conduct that is known or ought to be known to be unwelcome” [31]. The protected grounds include, among other things, age, disability, gender identity and expression, race and related grounds, and sexual orientation, with definitions for each derived from legislation and case law in Canada [31]. We are including 3 broad protected grounds in our analysis (Table 3), with the decision to exclude the remaining protected grounds because of the unreasonably high potential to identify study participants (eg, disability, age, citizenship, family status, marital status) or lack of plausibility (eg, record of criminal offenses, receipt of social assistance in housing).

**Table 3:** Included grounds with defining language drawn from the Ontario Human Rights Code.

Ground	Defining language
Gender identity and gender expression	Gender identity is each person’s internal and individual experience of gender. Gender expression is how a person publicly presents their gender.
Race and related grounds	Race is a social construct that includes geographic, historical, political, economic, social, and cultural factors, as well as physical traits. The code also includes ancestry, ethnicity, religion, and place of origin in this category.
Sexual orientation	Sexual orientation covers the range of human sexuality from lesbian and gay to bisexual and heterosexual.

With respect to potentially vexatious words, we will reference the definitions for each word (eg, fuck, bitch, cunt, fag, cock, etc) provided in the *Urban Dictionary* as a sensitizing concept to aid in the determination of the word’s potential to offend. The *Urban Dictionary* is a Wikipedia-style website that allows users to populate definitions for specific words based on contemporary usage in society [32].

To prepare the data, we will abstract the free-text incident narratives into a Microsoft Excel (Microsoft Corp) file for analysis. Drawing on the definitions for harassment and protected grounds provided in the code, we will use independent raters to assess the proportion of EVIRs that involve harassment on 1 or more protected grounds of interest. Specifically, we will recruit 2 research assistants with knowledge of the paramedic context and legal experience (ie, through university training or labor relations involvement) for this task. Following a period of rater calibration [27] with the research team, each rater will then independently evaluate each incident narrative to answer the following question: “Does the narrative suggest harassment on the basis of gender identity or expression, race and related grounds, or sexual orientation?” (Multimedia Appendix 3). We will calculate Cohen Kappa as a measure of interrater agreement, with a member of the research team resolving any areas of disagreement on a specific incident narrative.

Finally, for Research Question 3, we will examine the EVIR incident narratives for comments that refer to anger over COVID-19 health measures (ie, vaccination, masking, social distancing) that contribute to violent encounters. This might include, for example, hostile reactions in response to paramedics’ requests that patients or bystanders at scenes wear a mask. Again, we will abstract the EVIR incident narratives into a Microsoft Excel (Microsoft Corp) sheet and have 2 independent raters review each narrative to answer the following question: “Does the narrative suggest violence on the basis of conflict over COVID-19 health measures?” Using a similar approach to Research Question 2, we will engage in a period of rater calibration followed by independent coding and then use Cohen Kappa to measure interrater agreement.

All personnel recruited to analyze qualitative data will be required to undergo online research ethics training made available for free by the Government of Canada and be instructed to treat the data as strictly confidential. Because the incident narratives may include detailed descriptions of specific incidents that may later be identifiable to persons with knowledge of the events, we will not report direct quotes and will use general language in describing examples to support relevant themes during publication.

## Results

Although we originally planned to study a 1-year period, we have expanded our study window to include all violence reports filed between February 1, 2021 and February 28, 2023. As of January 1, 2023, a total of 975 EVIRs have been filed. The research program remains internally funded by the Region of Peel’s paramedic services division, and our analysis will begin when the data collection window closes on February 28, 2023. We anticipate the publication of several manuscripts blueprinted for each research objective in late 2023 through early 2024

We anticipate being able to deliver a detailed accounting of the prevalence of reported incidents of violence in the study site, as a proportion of both the total number of service calls and the number of active-duty paramedics in the service. Second, will compile a list of service call characteristics that suggest an increased risk of any form of violence and for violence resulting in an assault on a paramedic. Finally, we will generate a qualitative description of themes that underpin documented incidents of violence, including the potential contribution of violence based on gender, race and related grounds, sexual orientation, and conflict over COVID-19 public health measures.

## Discussion

### Overview and Strengths

Despite a growing recognition of the potential for significant physical and psychological harm [15,33-38], there is very little epidemiological data describing violence against paramedics [35,39]. Aside from some reviews of occupational injury statistics [34,37,40-43], much of the research on the topic has used cross-sectional survey approaches that ask paramedics about exposure to violence during the past 12 months or throughout their careers [14-16,33,35,39,44-49]. Although informative in drawing attention to the issue and demonstrating that a concerning majority of paramedics have been subjected to violence, the work leaves several important questions unanswered. Without robust, event-level reporting data, it is difficult to ascertain the scope of the problem and potentially modifiable risk factors—both of which are needed in developing organizational strategies to mitigate the risks and enhance paramedic safety.

The challenge in gathering robust data about violence is that the organizational culture within the profession has contributed to underreporting. When surveyed, many paramedics describe violence as just “part of the job” [14,15,17]. As a result, only a minority of incidents are reported to paramedic supervisors or the police. Our own work on the topic has demonstrated a cycle within which chronic exposure to violence, combined with its perceived inevitability and lack of consequences for perpetrators, serves to implicitly position the ability of paramedics to “brush off” violent encounters as an expected professional competency [17]. Breaking this cycle is an important step in understanding the scope of the problem. In that respect, our research has several strengths that will allow us to contribute meaningfully to this literature.

First, the reporting process was developed through an extensive stakeholder engagement and pilot testing process with the goal of promoting accessibility [19]. Embedding the EVIR within the patient care record gives us a streamlined, accessible, point-of-event reporting mechanism. This allows us to ask questions about the prevalence of violence as a proportion of both service calls and active-duty paramedics. We will also be able to generate a descriptive profile of the types of violence paramedics report, its sources, and contributing circumstances—an important first step to understanding the scope of the problem. Second, because the EVIRs are linked directly to ACRs, we will be able to evaluate the degree to which features of service calls that are generally known to the responding paramedics ahead of time (such as the nature of the problem) are associated with an increased risk of violence. This analysis can be used to develop strategies when responding to high-risk calls, such as coordinated response plans with police or mobile crisis teams. Third, qualitatively analyzing the free-text narratives potentially allows us to examine some of the root causes of violent encounters. It may be, for example, that violent encounters are precipitated by conflict over patient care, preferences, or psychosocial needs that can be better managed through improved policy or training. Relatedly, the degree to which paramedics are subjected to vitriol based on gender, race, or sexual orientation has not (to our knowledge) been previously studied and may be a novel contribution to the literature describing chronic stressors in paramedic work [50-52]. Examining the EVIR narratives for themes related to COVID-19 will generate modest empirical data in a local context on what media sources have been describing as a growing antipathy toward health care providers over public health measures, such as masking or vaccination mandates [18]. Finally, our analysis will enable refinements to the reporting mechanism itself. This can support both strengthened acceptance among the end users and scalability of the work to include other paramedic services in the province.

### Limitations

We are placing some important boundaries on this research that need to be considered as limitations when interpreting our findings. First, we must acknowledge that all findings to be shared from this research are limited to reported incidents from a single study site. Although the development phase went to great lengths to address many of the organizational cultural barriers to reporting, we are mindful that the work is coming up against a very real and historically entrenched reluctance to report violent incidents. The degree of underreporting we will observe in our study is difficult to estimate. Both factors limit the generalizability of our findings. Second, we are gathering data about the incidents themselves but stopping short of evaluating the health consequences for the reporting paramedics in terms of physical injuries, lost time from work, or more nebulous psychosocial harms, such as burnout. Given the links between critical (or traumatic) stress and PTSI in the paramedic population, studying the impact on health and well-being will be an important next step in the research. Third, our statistical analyses will necessarily be constrained by the observed event rate, which is difficult to estimate ahead of time. Our evaluative time frame of 1 year following implementation is admittedly arbitrary, but it is reasonable from a feasibility perspective and aligns with the operational needs and priorities of the paramedic service.

### Conclusion

Leveraging a novel, point-of-event reporting process, this study aims to describe the prevalence of violence against paramedics in the Peel Region, its risk factors, and underpinning themes. This research will broadly be used to develop evidence-informed risk mitigation strategies, support a wider-scale implementation of violence tracking, and contribute epidemiological data to what has been described as a “serious public health issue” [15].

## Appendix

Multimedia Appendix 1 External Violence Incident Report (EVIR).

Multimedia Appendix 2 Data points to be analyzed as potential covariates from the ambulance call report.

Multimedia Appendix 3 Template for independent coding of incident report narratives for Objective 3, Research Question 2.

## Abbreviations

ACP: advanced care paramedic
ACR: Ambulance Call Report
AOR: adjusted odds ratio
ePCR: electronic patient care record
EVIR: External Violence Incident Report
PCP: primary care paramedic
PRPS: Peel Regional Paramedic Services
PSP: public safety personnel
PTSI: posttraumatic stress injury
PTSD: posttraumatic stress disorder

## Data Availability

Limited quantitative data from this study can be made available to interested parties upon request from the principal investigator. Note that this may require a formal data-sharing agreement.
