# Supplementary figures and images for: Violence Against Paramedics: Protocol for Evaluating 2 Years of Reports Through a Novel, Point-of-Event Reporting Process
Source: JMIR Res Protoc. 2023 Mar 16;12:e37636. doi: 10.2196/37636 (PMC10131719; doi:10.2196/37636)

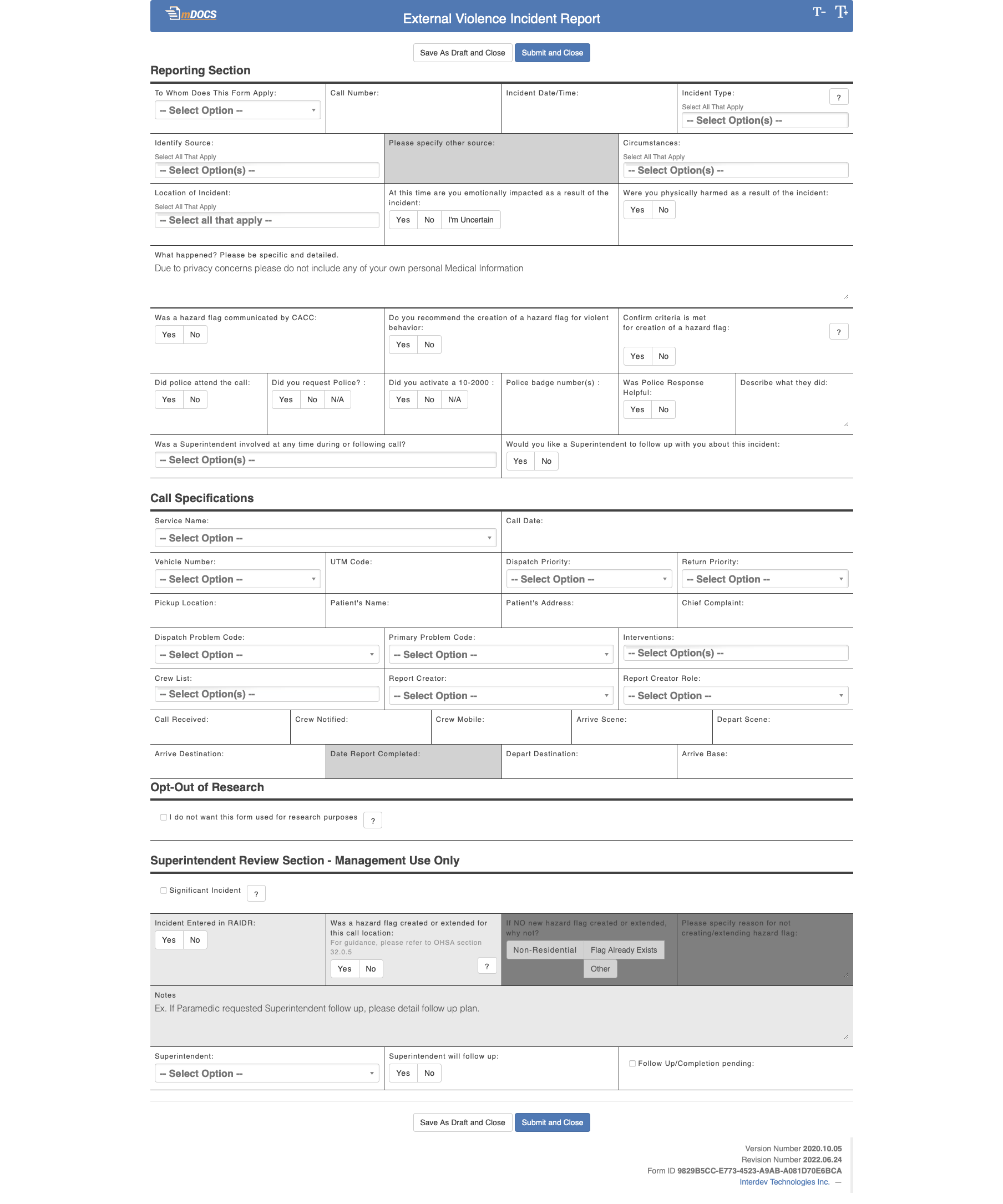

Supplement: Multimedia Appendix 1 [file resprot_v12i1e37636_app1.png]
