# Supplementary material for: Violence Against Paramedics: Protocol for Evaluating 2 Years of Reports Through a Novel, Point-of-Event Reporting Process
Source: JMIR Res Protoc. 2023 Mar 16;12:e37636. doi: 10.2196/37636 (PMC10131719; doi:10.2196/37636)
Supplement: Multimedia Appendix 2 [file resprot_v12i1e37636_app2.docx]

| **Paramedic Station of Origin** | Fernforest Division  Rising Hill Division  Tomken Division  Streetsville Division | **Dispatch / Primary Problem Codes** | |
| --- | --- | --- | --- |
|  |  | **Vital Signs Absent (VSA)**  01 - Cardiac / Medical  02 - Traumatic  **Airway**  11 - Obstruction  **Breathing**  21 - Dyspnea  24 - Respiratory Arrest  **Circulation**  31 - Hemorrhage  33 - Hypotension  34 - Sepsis  **Neurological**  40 - Traumatic Brain Injury  41 - Stroke  42 - Temp. Loss of Consciousness  43 - Altered Level of Consciousness  44 - Headache  45 - Behavior / Psychiatric  45.01 - Excited Delirium  46 - Active Seizure  47 - Paralysis / Spinal Trauma  48 - Confusion / Disorientation  49 - Unconscious  50 - Post-ictal  **Cardiac**  51 - Ischemic (chest pain)  53 - Palpitations  54 - Pulmonary Edema  55 - Post Arrest  56 - Cardiogenic Shock  57 - STEMI  58 - Hyperkalemia  **Non-Traumatic**  60 - Non-Ischemic Chest Pain  61 - Abdominal Pain  62 - Back Pain  63 - Nausea / Vomiting  **Musculoskeletal**  66 - Musculoskeletal  67 - Trauma  **Obstetrics/Gynecological**  71 - Obstetrical Emergency  72 - Gynecological Emergency  73 - Newborn  **Endocrine/Toxicological**  81 - Drug / Alcohol | 81.1 - Opioid Overdose  82 - Poisoning / Toxic Exposure  83 - Diabetic Emergency  84 - Allergic Reaction  86 - Adrenal Crisis  **General and Minor**  87 - Novel Medications  88 - Home Medical Technology  89 - Lift Assist  90 - Inter-facility Transfer  91 - Environmental Emergency  92 - Weakness / Dizziness / Unwell  93 - Treatment / Diagnostic / Return  94 - Convalescent  95 - Infectious Disease  96 - Organ Retrieval  98 - Organ Recipient  99 - Other |
| **Date** | YYYY-MM-DD |  |  |
| **Call Received Time** | Time call received at the Central Ambulance Communications Center |  |  |
| **Pick up Location Code** | A - Airport/Heliport  B - Apartment / Condo Building  C - Construction Site  D - Medical Office / Clinic  E - Nursing Outpost  F - Factory  G - Hotel  H - Hospital  I - Indoor Shopping Mall  J - Jail / Prison  K - Single Store  L - School / College / University  M - Mining Site  N - Long-Term Care Home  O - Office Building  P - Sports Facility  Q - Farm  R - House  S - Street / Highway / Road  T - Fairground / Park  U - Retirement Home  V - Golf Course  W - Water / Boat  X - Restaurant / Bar  Y - Casino  Z - Other |  |  |
| **Dispatch / Return Priority** | 1 - Deferrable  2 - Scheduled  3 - Prompt  4 - Urgent |  |  |
| **Return Priority (Without Patient)** | 71 - No Patient Found  72 - Patient Refused  73 - Patient Deceased  74 - Patient in Police Custody  75 - Transported by Other Ambulance  77 - Treated and Released |  |  |
