# Supplementary material for: Violence Against Paramedics: Protocol for Evaluating 2 Years of Reports Through a Novel, Point-of-Event Reporting Process
Source: JMIR Res Protoc. 2023 Mar 16;12:e37636. doi: 10.2196/37636 (PMC10131719; doi:10.2196/37636)
Supplement: Multimedia Appendix 3 [file resprot_v12i1e37636_app3.docx]

Template for independent coding of incident report narratives for Objective 3, Research Question 2.

| **Case** | **Text** | **Does the narrative suggest harassment on the basis of:** | | |
| --- | --- | --- | --- | --- |
|  |  | Gender Identity or Expression | Race or Related Grounds | Sexual Orientation |
| 1 |  |  |  |  |
